# Foetal Mummification in Pregnant Dairy Cows Induces Variant Changes on the Hormonal Profile, Biochemical Parameters and Mineral Profile of the Dam

**DOI:** 10.1002/vms3.70304

**Published:** 2025-04-25

**Authors:** Yahia A. Amin, Obeid Shanab, Ibrahim S. Zahran, Foad Farrag, Mariam A. Fawy, Mustafa Shukry, Mohamed Abdelmegeid, Eman M. Abu El‐Naga, Ragab H. Mohamed, Ahmed A. Elolimy

**Affiliations:** ^1^ Department of Theriogenology Faculty of Veterinary Medicine Aswan University Aswan Egypt; ^2^ Department of Biochemistry Faculty of Veterinary Medicine South Valley University Qena Egypt; ^3^ Department of Physiology Faculty of Veterinary Medicine Aswan University Aswan Egypt; ^4^ Department of Anatomy and Embryology Faculty of Veterinary Medicine Kafrelsheikh University Kafrelsheikh Egypt; ^5^ Department of Zoology Faculty of Science South Valley University Qena Egypt; ^6^ Department of Physiology Faculty of Veterinary Medicine Kafrelsheikh University Kafrelsheikh Egypt; ^7^ Veterinary Program Faculty of Health Sciences Higher Colleges of Technology Sharjah Men's Campus Sharjah UAE; ^8^ Department of Internal Medicine Faculty of Veterinary Medicine Kafrelsheikh University Kafrelsheikh Egypt; ^9^ Department of Integrative Agriculture College of Agriculture and Veterinary Medicine United Arab Emirates University, Al Ain Abu Dhabi UAE

**Keywords:** biological activities, cows, foetus, mummification of the foetus, normal pregnancy

## Abstract

**Background and aim:**

Foetal mummification does not cause loss by losing foetus only but the harm extends to the mother dam. This is the first study that investigates the changes that occur in the dam's body due to foetal mummification through a comprehensive investigation of the hormonal, biochemical, liver, kidney and mineral profiles of the dam. This study represents a model to investigate the pathogenesis of such clinically severe cases.

**Materials and methods:**

This study involved 40 cows with foetal mummification. The dams were divided into 2 groups (20 for each): Group 1 (cows carrying normal foetuses [CNF]) and Group 2 (cows carrying mummified foetuses [CMF]). Blood samples were collected for evaluation of hormonal profile, biochemical profile, kidney profile, liver profile and mineral profile.

**Results:**

Results showed that progesterone (P4) and oestradiol (E2) concentrations were not significantly changed in the CMF group compared to the CNF group, whereas total triiodothyronine (T3) and total thyroxin (T4) showed a significant reduction in mummified cases than in CNF group. In the CMF group, parameters such as glucose and albumin were significantly lower compared to the normal pregnant animals. In contrast, triglycerides, cholesterol, globulin and total protein (TP) were significantly higher. Kidney and liver profiles showed a significant increase in urea, aspartate aminotransferase (AST), alkaline phosphatase (ALP) and bilirubin, a significant decrease in alanine aminotransferase (ALT) and non‐significant differences in creatinine and gamma‐glutamyl transpeptidase (GGT) in CMF compared with normal pregnant cows. Mineral profiles showed a significant decrease in calcium and phosphorus, a significant increase in iron and potassium and a non‐significant increase in magnesium, sodium and chloride in the mummified group compared to the normal pregnancy group.

**Conclusion:**

Cows with mummified foetuses (MF) maintained variant biological changes in the body. These findings can be used as an indicator for cow health and as a diagnostic tool to avoid pregnancy disorders that occur during the late pregnancy.

## Graphical abstract

A comprehensive investigation was conducted on the hormonal, biochemical, liver, kidney and mineral profiles of pregnant cows carrying mummified foetuses. The results showed a significant increase in glucose, albumin, triglycerides, cholesterol, globulin, total protein, urea, AST, ALP, bilirubin, iron and potassium in cows carrying mummified foetuses, compared to a significant decrease in T3, T4, ALT, calcium and phosphorus. These findings provide valuable indicators of cow health and serve as diagnostic tools for pregnancy disorders during late pregnancy.

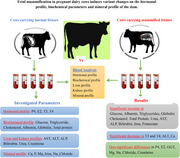

## 1 Introduction

Pregnancy losses in dairy cattle represent one of the most difficult problems that cause high financial losses. Several studies of dairy cattle have illustrated that the economic loss due to pregnancy loss can vary from US$90 to $1900, based on the gestational stage at which the pregnancy was lost. This cost is linked to several factors, such as a longer time elapsed between calvings, fewer possible replacement heifers being available, lower milk production, higher insemination costs, veterinary and labour expenses and premature culling (De Vries 2006; Cabrera 2014). Foetal loss can result from a variety of causes, such as genetics, toxic substances (Yahia et al. 2020), infectious diseases and heat stress. One of the most significant types of pregnancy losses during the foetal stage is a mummified foetus (MF). Foetal mummification rates in cows range from 0.13% to 1.8% (Alok and Atul 2018). Foetal mummification is occasionally diagnosed in different animal species, such as cattle (Shah et al. 2018), buffalo (Kumar et al. 2019) and goats (Bisla et al. 2018).

Mummification cases of pregnant animals are marked by a reduction in foetal fluid and the presence of brown debris encircling the foetus (Kumar et al. 2017). Foetal mummification can occur between 3 and 8 months of gestation, following the development of the placenta and the foetus's bones, and without cervical dilatation or contamination that would have resulted in luteolysis (Alok and Atul 2018; Krishan 2015). Following foetal mummification, the corpus luteum continues to release progesterone (Alok and Atul 2018). Foetal mummification is characterized by the hard foetal body inside the uterine horns without any clinical signs, the failure of involution of the foetal and maternal parts of the placenta, and the difficulty of expulsing a dead foetus (Krishan 2015). The hypothesis on the aetiology of intrauterine foetal mummification in cases of foetal mummification due to non‐infectious disorders is that the foetus and the foetal membrane undergo dehydration, which neutralizes tissue autolysis in the absence of oxygen and bacteria (Alok and Atul 2018). Haematic mummification is the type that occurs in cows most frequently and may later develop into foetus maceration (Kumar et al. 2017).

Blood biochemical parameter analysis can be used to assess the nutritional and physiological health of cows (Ashmawy 2015). Inconsistencies in many biochemical factors have been implicated in making cows suffer from an inability to do the reproduction process (Bazzano et al. 2016). Assessment of blood markers such as glucose (GLU), proteins, urea and non‐esterified fatty acids could be used as indicators for animal's energy, health and nutritional state (Xuan et al. 2018).

The current study hypothesizes that mummification of the foetus induces different biological changes that occur in the body of the pregnant mother. Therefore, the objectives of this study are to characterize the early alterations in the hormonal profile (progesterone, oestradiol, T3 and T4), biochemical profile (GLU, TGs, cholesterol, albumin, globulin and total protein [TP]), kidney profile (urea, creatinine), liver profile (aspartate aminotransferase [AST], alkaline phosphatase [ALP], bilirubin, alanine aminotransferase [ALT] and gamma‐glutamyl transpeptidase [GGT]), as well as mineral profile (calcium, phosphorus, iron, magnesium, sodium, potassium and chloride) of dairy cows suffered from foetal mummification and to search for early screening biomarkers that can be used as a predictive biomarkers for mummification of the foetus. The detection of the circumstances associated with foetal mummification can aid scientists in comprehending the aetiology and clinical state of this case in various species.

## 2 Materials and Methods

### 2.1 Animals

The present study was conducted on local breed cows from a farm in Qena province in the south of Egypt. All the data on the reproductive history of the animals (oestrous cycle, calving condition, postpartum period [PPP] and uterine involution) were known. The cows were divided into two groups: Group 1 (cows carrying mummified foetuses [CMF], *n* = 20) and Group 2 (cows carrying normal foetuses [CNF], *n* = 20). The case history of the animals revealed that the cows were fed using a total mixed ration (TMR) system twice a day and milked twice a day. The NRC guidelines were followed in the preparation of the TMR diet's chemical components. Besides, water is available to animals indefinitely. Cows were apparently healthy with average age of 4–5 years and average parity of 2.5 ± 0.4, whereas the mean weight was 320 ± 0.15 kg, and body condition score (BCS) was ≥3. The detection of BCS depends on a five‐point scale of one (thin) to five (obese).

The breeding system and management of the farm are based on applying synchronization with controlled internal drug release (CIDR) and prostaglandin F2α (PGF2α) (Chebel et al. 2010; Amin et al. 2019). The animals selected were expected to have pregnancy at the same time and age. The animals were examined by a local veterinarian at 90 days after natural serving, and the animals were found pregnant. The animals were apparently healthy; the feed intake was normal, and clinical parameters, including temperature, heart rate and respiration rate, were within normal limits. The herd in the farm undergoes regular vaccination against the most common infectious diseases causing abortion and other reproductive problem. There were no previous pregnancy‐related complications with the dams in the mummified group. There were no visual signs approaching parturition.

Diagnosis of mummification of the foetus occurred by two different routes: per rectal palpation and ultrasonographic examination (Pie Medical, 100 LC, Maastricht, the Netherlands) using a sonar device connected to a 6/8 MHz changeable transducer. A compact, hard and immobile mass lacking foetal fluid, or placentomes, appears to be the most common finding in cases of MF, according to rectal palpation and ultrasonographic examination. The cervix was found to be closed with no discharge. With the exception of a few uncommon instances where a reduction in milk supply and weight loss has been seen, the dam's overall physical assessment seems normal (Frazer 2004).

Following confirmation of pregnancy that occurred by the veterinarian at 90 days, examination of the animal during the periodic examination (every 2 months) revealed the presence of this condition (cows carrying MF) at the age of more than 90 days of pregnancy (average of 5 months). As synchronization protocol was usually used during breeding management of the farm, the 20 cows of the MF group showed mummification at the same time. Table 1 shows details of the two groups of cows that carry MF and/or NF (first diagnosed with the case, the month of pregnancy and the age of the MF at diagnosis).

**TABLE 1:** Details of the two groups of cows that carrying mummified foetuses (MF) and/or normal foetuses (NF), first diagnose with the case, the month of pregnancy and age of the MF at diagnosis.

Groups	Time of first diagnosis of pregnancy	Time of first diagnosis of MF	Age of MF at diagnosis
Cows carrying MF	90 ± 1.58 days	Second periodic examination showed MF	Ages of MF were 150 ± 1.55 days
Cows carrying NF	90 ± 1.78 days	Second periodic examination showed NF	Ages of NF were 150 ± 1.68 days

*Note*: Values are mean ± SE.

The treatment of MF cases was performed with a PGF2α injection; however, one case of maceration was observed which indicates that this case was not mummified but it was macerated from the beginning. One cow was treated by hysterectomy due to a failure of medical treatment.

### 2.2 Collection of Blood Samples

The jugular vein was used for the collection of blood samples, which occurred by using disposable plastic syringes at the following days: Day 0 (day of detection of mummification of the foetus), Days 15 and 30 (after detection of mummification of the foetus) for the cows of the MF group and on the same days for the control normal foetus cows group (5 months pregnant cows). Ten millilitres of blood was drawn, and 2 mL of that blood was immediately transferred to a test tube containing fluorine oxalate, an anticoagulant, so that the blood GLU could be measured. The remaining blood samples were moved to a plain tube, allowed to coagulate naturally at ambient temperature (28°C) and then centrifuged for 10 min at 2000 rpm. After separating the serum samples, they were put into sterile plastic vials and promptly frozen at −20°C in preparation for further analysis.

### 2.3 Biochemical and Hormonal Analysis

Using the progesterone (P4) ELISA Kit (Catalogue No.: E‐OSEL‐B0005) from Elabscience, USA, serum P4 concentrations were measured. Following the manufacturer's instructions, serum was tested using the oestradiol (E2) ELISA Kit product from Elabscience, USA (Catalogue No.: E‐OSEL‐B0001). Total triiodothyronine (T3) and total thyroxin (T4) were assayed using ELISA kits (Monobind Inc., T3 AccuBind ELISA Kits, and T4 AccuBind ELISA Kits, USA). The Erba biochemical kit (ERBA Mannheim, Erba Lachema) was used to analyse glucose (GLU). As soon as blood was drawn, the blood GLU level was ascertained. Triglyceride (TG) GPO‐POD Liquid Kits (Spinreact) were used for the evaluation of TG. Measuring of cholesterol, albumin and total protein (TP) was performed using kits from Biodiagnostic through the enzymatic colorimetric method, the colorimetric method and the protein biuret method, respectively. The difference between the concentrations of TP and albumin was used to calculate globulin.

As directed by the manufacturer, blood urea nitrogen (BUN) was detected using kits from Spectrum by applying the Berthelot method, whereas creatinine was detected using kits from Biodiagnostic using the colorimetric method.

Alanine aminotransferase (ALT), Aspartate aminotransferase (AST) and Alkaline phosphatase (ALP) activities were detected by the colorimetric method using kits from Biodiagnostic. Total bilirubin (TBIL) was measured by an automatic biochemistry analyser (AU480, Beckman Coulter, USA) using commercial kits (Ningbo Purebio Biotechnology Co. Ltd.). The concentration of Gamma‐glutamyl transpeptidase (GGT) was determined using commercial kits of the Randox Laboratories brand in an automatic biochemistry system (RX Daytona; Randox Laboratories).

The A25‐Biosystem‐Random Access Analyser, a fully automated analyser, was used to examine the levels of calcium (Ca), phosphorus (P), magnesium (Mg) and iron (Fe) in plasma using commercial kits (BioSystems, S.A., Barcelona, Spain). Sodium (Na) and potassium (K) were detected using kits from Biodiagnostic through the colorimetric method and the turbidimetric method, respectively. Using a semi‐automated clinical chemistry analyser (AGAPPE Diagnostics, India), the Erba biochemical kit (BLT00033) was used to test chloride (CL).

### 2.4 Statistical Analysis

The data obtained were analysed statistically with the help of SPSS version 20 (IBM Corp., NY, USA) software using one‐way ANOVA followed by Duncan's multiple range tests. Normality test was done using the Kolmogorov–Smirnov test, and repeated measures ANOVA with Bonferroni post hoc test were performed. The data are expressed as mean ± SE with a significance level of *p* ≤ 0.05.

## 3 Results

**FIGURE 1:**
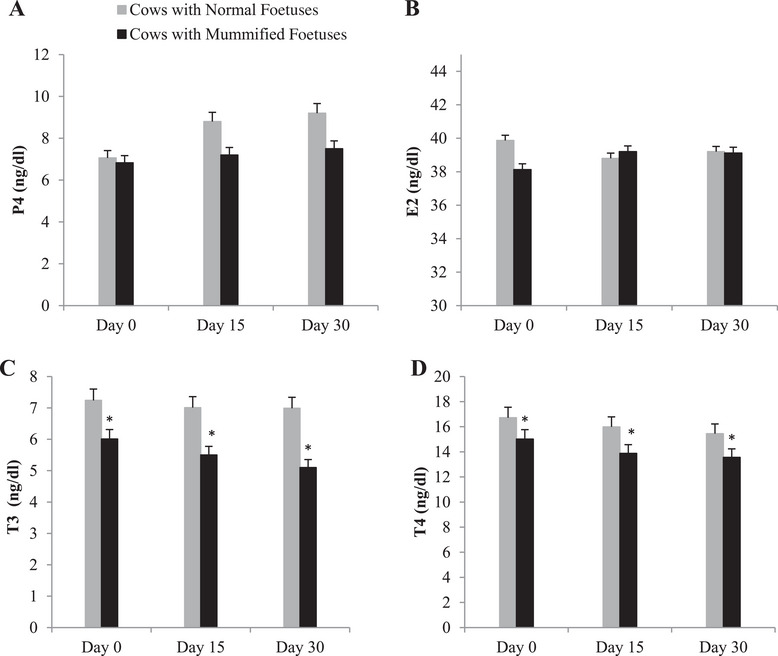
Summed changes (mean ± SE) in (A) progesterone hormone (P4), (B) oestradiol hormone (E2), (C) T3 hormone, (D) T4 hormone levels in serum of cows with mummified foetuses (*n* = 20) and control cows with normal foetuses (*n* = 20) at the Day 0 (the day of detection of fetal mummification), Days 15 and 30 (after detection of mummification of the foetus). Asterisks (*) indicate statistically significant (*p* < 0.05) difference between cows with mummified foetuses and control cows with normal foetuses at the same time point.

The animal appears to be healthy but has suffered from reduced feed intake and a drop in milk production. Progesterone and E2 concentrations are not significantly different in the group of MFs of cows compared to the normal pregnant group of the same animals (Figure 1A,B, respectively). The levels of T3 and T4 (ng/dL) are significantly decreased in mummified cases than in normal pregnancy cases (Figure 1C,D, respectively).

**FIGURE 2:**
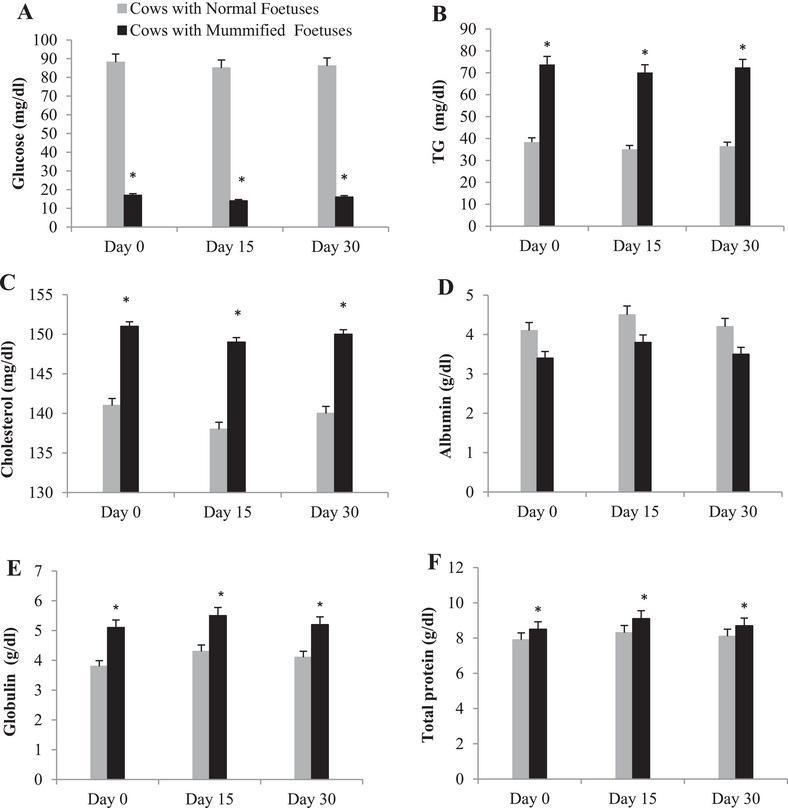
Summed changes (mean ± SE) in (A) glucose (GLU), (B) triglyceride (TG), (C) cholesterol, (D) albumin, (E) globulin and (F) total protein levels in serum of cows with mummified foetuses (*n* = 20) and control cows with normal foetuses (*n* = 20) at the Day 0 (day of detection of mummification of the foetus), Days 15 and 30 (after detection of mummification of the foetus). Asterisks (*) indicate statistically significant (*p* < 0.05) difference between cows with mummified foetuses and control cows with normal foetuses at the same time point.

Blood GLU (mg/dL) levels in the normal pregnant animals were within the normal range or slightly higher (40–60). In contrast, the cows of the MF group exhibited a significant decrease (*p* < 0.05) in mean blood GLU concentrations in all time points than the normal pregnant animals (Figure 2A). Triglycerides and cholesterol levels in the group of MF cows were significantly increased in all time points compared to their levels in normal pregnant animals (Figure 2B,C, respectively). Serum albumin detection reveals non‐significant reduction in its levels in the different time points in cows with MFs relative to healthy cows (Figure 2D). Conversely, globulin and TP were found to be significantly decreased in normal pregnant cows in the studied time points compared to those with MF cows (Figure 2E,F, respectively).

**FIGURE 3:**
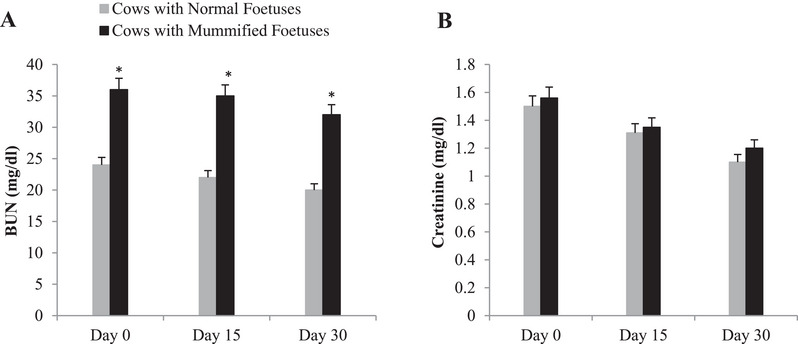
Summed changes (mean ± SE) in (A) blood urea nitrogen (BUN), and (B) creatinine levels in serum of cows with mummified foetuses (*n* = 20) and control cows with normal foetuses (*n* = 20) at Day 0 (day of detection of mummification of the foetus), Days 15 and 30 (after detection of mummification of the foetus). Asterisks (*) indicate statistically significant (*p* < 0.05) difference between cows with mummified foetuses and control cows with normal foetuses at the same time point.

A significant increase in the mean BUN level was found in cows with MFs compared with normal pregnant cows in the same evaluated time points (Figure 3A). In contrast, a non‐significant increase was observed in creatinine concentrations of cows with an MF compared to normal pregnant cows (Figure 3B).

**FIGURE 4:**
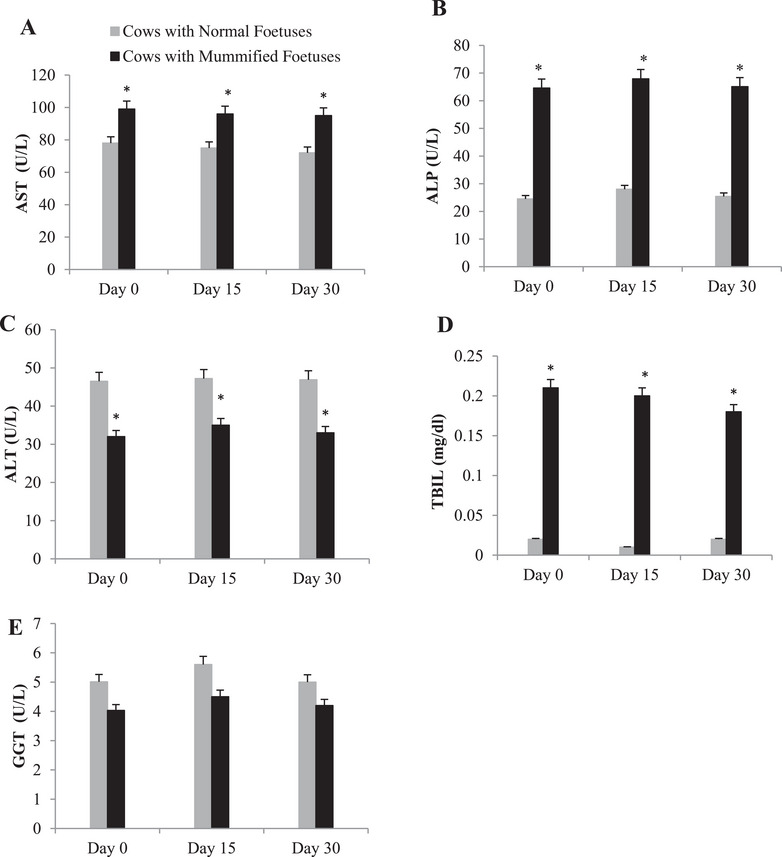
Summed changes (mean ± SE) in (A) aspartate aminotransferase (AST) activity, (B) alkaline phosphatase (ALP), (C) alanine aminotransferase (ALT) activity, (D) total bilirubin (TBIL) and (E) gamma‐glutamyl transpeptidase (GGT) levels in serum of cows with mummified foetuses (*n* = 20) and control cows with normal foetuses (*n* = 20) at the Day 0 (day of detection of mummification of the foetus), Days 15 and 30 (after detection of mummification of the foetus). Asterisks (*) indicate statistically significant (*p* < 0.05) difference between cows with mummified foetuses and control cows with normal foetuses at the same time point.

Liver indicators such as AST, ALP and TBIL were found to be significantly increased in the MF group compared to normal pregnant ones (Figure 4A,B,D, respectively) in all time points. On the contrary, ALT was found to be significantly decreased in the mummified group compared to normal pregnant group (Figure 4C). Furthermore, no difference was discovered in serum GGT concentration between cows with and without an MF (Figure 4E).

**FIGURE 5:**
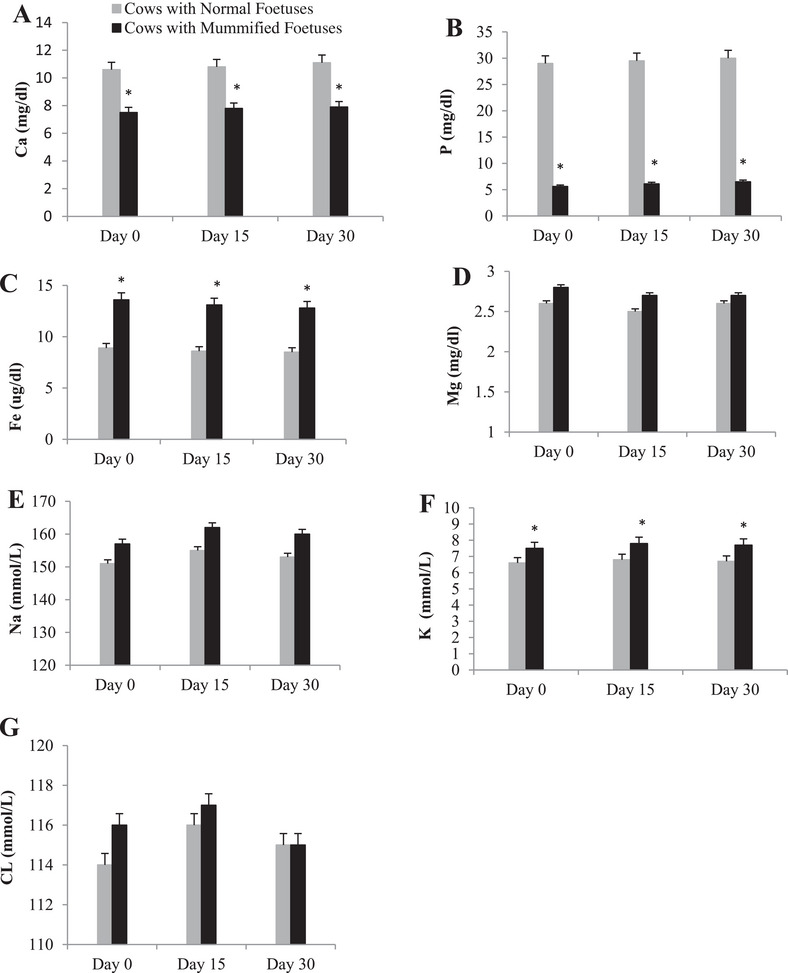
Summed changes (mean ± Se) in (A) calcium (Ca), (B) phosphorus (P), (C) iron (Fe), (D) magnesium (Mg), (E) sodium (Na), (F) potassium (K) and (G) chloride (CL) concentrations in serum of cows with mummified foetuses (*n* = 20) and control cows with normal foetuses (*n* = 20) at the Day 0 (day of detection of mummification of the foetus), Days 15 and 30 (after detection of mummification of the foetus). Asterisks (*) indicate statistically significant (*p* < 0.05) difference between cows with mummified foetuses and control cows with normal foetuses at the same time point.

Calcium and phosphorus concentrations were found significantly decreased in the studied time points in cows with an MF compared with normal pregnant cows (Figure 5A,B, respectively). Iron and Mg levels were found increased in cows with an MF than in normal pregnant cows, but this increase was significant in the case of iron and not significant in the case of Mg (Figure 5C,D, respectively). For Na and chloride, results revealed that elements were found non‐significantly increased in the mummified group than in the normal pregnancy group in the three evaluated time points (Figure 5E,G, respectively). Our results on mummified group showed a significant increase in potassium concentration than in normal pregnant group, and its levels were higher than the normal range (Figure 5F).

## 4 Discussion

Progesterone is one of the most significant hormones that is mainly engaged in receptor coordination of female reproductive processes, including the start and maintenance of pregnancy (De Amicis et al. 2012). Therefore, pregnancy maintenance and uterine preparation are among P4's functional characteristics (Blavy et al. 2016), and a low progesterone level is responsible for embryo loss as recorded in aborted cases of dairy cows (Amin, Omran et al. 2023). In the current study, P4 and E2 concentrations were not significantly different in the group of MFs of cows compared to the normal pregnant group of the same animals.

The similarity in high P4 concentration between animals suffering from mummification of the foetus and normal pregnant ones gives an explanation for the failure of the animals suffering from mummification of the foetus from expulsion of the dead foetus outside the uterus. The non‐significant difference of P4 in the MF group may be attributed to the presence of corpus luteum that failed to regress after the death of the foetus, which imitated a condition similar to that of the normal pregnant cows. The findings in the current study are somewhat similar to the other studies concerned with dystocia and retained placenta in cows. During pregnancy, P4 levels can range from low (6–15 ng/ml) to high (20–50 ng/ml). In cows with retained placental membranes, there is a decrease in oestradiol and a rise in serum P4 (Kaczmarowski et al. 2006; Amin, Elqashmary et al. 2023). The incidence of dystocia, or difficult calving, in cows has been associated with changes in the P4 profile and decreased production of oestrone sulphate (Kindahl et al. 2004). Similarly, recent research in buffaloes suffered from dystocia mentioned decreased level of E2 in the foetal fluid of dystocia cases compared to normal delivered cases (Amin et al. 2020).

In the present study, the levels of T3 and T4 were lower in mummified cases than in normal pregnancy cases. These findings somewhat concur with earlier research that found lower T3 and T4 levels in the post‐calving compared to the pre‐calving periods (Fiore et al. 2015; Djoković et al. 2015). The decreased level of T3 could perhaps be attributed to negative energy balance (NEB) conditions, which are marked by a greater mobilization of non‐esterified fatty acids from body stores during the postpartum phase (Djoković et al. 2015).

Blood GLU is one of the primary regulators of blood hormones and metabolites; thus, it is considered one of the factors that control reproduction. In dairy cattle, proper functioning of the ovaries and uterus requires adequate levels of blood GLU. In this study, blood GLU levels in the normal pregnant animals were within the normal range of 40–60 mg/dL (Mair et al. 2016) and may be slightly higher. In contrast, the cows of MF group have significantly (*p* < 0.05) lower GLU concentrations than the normal pregnant animals. These results are in line with the previous studies. Mohammed et al. (2021) indicate that the concentration of blood GLU was higher during late pregnancy and in the PPP than in non‐pregnant cows. According to research by Kalasariya et al. (2017), the mean blood GLU levels increased with increasing gestation starting on Day 60 prepartum and peaked on the day of delivery. Because the placenta and foetus are unable to store GLU against a concentration gradient, lower maternal GLU results in lower foetal GLU levels. Moreover, gluconeogenesis in the foetus and placenta is limited (Battaglia and Meschia 1978). Because pregnancy depends on GLU as a substrate for tissue synthesis and metabolic energy, a decrease in the amount of GLU that reaches the uterus and conceptus may have an impact on how the pregnancy develops (Battaglia and Meschia 1978). Thus, the growth of the foetus and placenta is dependent upon the metabolic environment of cows. Low GLU levels in postpartum cows may put them at risk of pregnancy loss because the placenta and foetus may not have enough substrate to grow new cells. Therefore, these findings encourage the suggestion that low maternal GLU levels may play a role in the occurrence of foetal mummification in pregnant cows and buffaloes.

In the present study, serum TG levels in the group of MF cows were significantly higher than in normal pregnant animals. This would be attributed to high fat mobilization in mummified group. These findings are similar to those previously mentioned in previous trials that suggested higher levels of TG in cows suffering from metritis, retained placenta, and pyometra are a possible result of increased energy needs (Seifi, Gorji‐Dooz et al. 2007; Seifi, Dalir‐Naghadeh et al. 2007; Amin et al. 2021).

Cholesterol concentrations were higher in the group of mummified cows than in normal pregnant animals. Lipoproteins are composed of apoproteins (apo), TG, cholesterol (C) and phospholipids (PL). During the induced NEB at around 100 days in milk (DIM), PL and cholesterol plasma concentrations were elevated in feed‐restricted (FRES) cows (Kessler et al. 2014).

In a study using an NEB during various lactation phases, Bjerre‐Harpøth et al. (2012) corroborated the rise in cholesterol concentration but did not find any changes in PL concentrations. These authors proposed that the increased hepatic output of very low‐density lipoproteins (VLDL) was the cause of the raised plasma cholesterol concentrations. Additionally, it was thought that the release of VLDL might be restricted by a reduction in hepatic PL synthesis (Van Den Top et al. 1996). Gross et al. (2015) showed that plasma concentrations of total cholesterol, PL, triglycerides (TG), very low‐density lipoprotein‐cholesterol (VLDL‐C) and low‐density lipoprotein cholesterol (LDL‐C) increased in feed‐restricted (FRES) cows from Week 0 to 3 during feed restriction compared to control cows. This indicates that the group of cows with foetal mummification suffers from NEB.

Another point of view is that, as a precursor to the steroid hormones (E2 and P4) made in the ovaries, cholesterol takes part in the process of steroidogenesis (Yart et al. 2014). On the basis of ovarian steroid hormones, P4 suppresses immunity, whereas E2 may strengthen immunity and help get rid of microorganisms in the uterus (Lewis 2003). During the puerperium, cows with elevated cholesterol levels will generate higher levels of ovarian steroid hormones (Teixeira et al. 2017). This may give an explanation for the increased concentration of cholesterol in group of foetal mummification.

Our results revealed a reduction in the levels of albumin in cows with MFs relative to healthy cows. Similar results were reported in cases of cows suffering from either placental retention and/or endometritis, in which lower levels of albumin were observed in these diseased cases compared to cows with physiological puerperium and/or those free from endometritis (Saut et al. 2014; Souza et al. 2010). According to Tóthová et al. (2013), albumin is a significant negative acute phase protein that is generated in the liver and is in charge of transporting molecules as well as controlling plasma osmotic pressure. Because of this, cows that had an MF had reduced levels of albumin, which is produced by the liver. This could be because of an NEB. In addition, an increased level of albumin in normal pregnant cows may be due to its need later for parturition. According to Piccione et al. (2011), there is an increase in albumin concentration during parturition. This increase may be caused by the liver producing more albumins or by a drop in plasma volume that is hidden by hypoglobulinemia. Furthermore, fatty liver, a common condition in the early stages of lactation, can also have an impact on serum albumin concentration (Sevinc et al. 2001). Nevertheless, certain liver function indicators were not examined in the current investigation.

Conversely, globulin in the current study was found to be low in normal pregnant cows compared to those with MFs. Previously, it was reported that γ‐globulins are transported from the blood to the colostrum; therefore, normal pregnant cows’ plasma typically has low levels of globulin (Weaver et al. 2000). In addition, Larson and Kendall (1957) illustrated that before parturition, the excretion of β2‐ and γ1‐globulins in colostrum causes a 10%–30% drop in plasma protein concentrations in dairy cows.

Elzein et al. (2016) reported similar protein concentration results in dairy goats at the end of gestation, during parturition and after the animals returned to normal in the PPP. Additionally, the authors demonstrated that globulin migration toward colostrum production may be the cause of this (Anwar et al. 2012). In another study, it was stated that as a physiological reaction to parturition, serum globulin was elevated at the start of lactation (maybe because of an increase in α‐globulins), and it declined linearly as DIM advanced (Bobbo et al. 2017).

The TP concentration in the current study was found low in normal pregnant cows compared to those with MFs. This outcome is consistent with the findings of another study that showed a drop in serum TP throughout late pregnancy (Kurpinska et al. 2015). This suggests that there was a significant change in serum TP levels from the physiological phase (Piccione et al. 2012). The cow undergoes significant metabolic stress during the gestation period, which causes this shift in protein content (Piccione et al. 2011). Moreover, it was stated that reduced TP levels were found around parturition (Blum et al. 1983); however, as albumin did not alter considerably, the reduction in TP concentrations was caused by a decrease in globulin, which was lost in the colostrum. TP increased quickly at the start of lactation, peaked in quantities between 30 and 100 days later and then gradually declined. Thus, as confirmed by our results, the fluctuation in TP concentration between cows with normal pregnancy and those with MFs returns to the need of a normal pregnant cow for high protein content for later parturition and lactation. Moreover, the most significant factors were found to have an influence on the serum TP, globulin concentration and albumin‐to‐globulin ratio (A:G) involved in the stages of gestation and lactation (Piccione et al. 2011). This influence was especially noticeable during the shift from late gestation to early lactation, when cows were forced to face significant metabolic stress.

In the present study, a higher mean urea level was found in cows with MFs compared with normal pregnant cows. Likewise, recent trials stated similar results in which higher urea levels were found in dairy cows suffering from pyometra and/or postpartum metritis compared to normal, healthy cows (Amin et al. 2021; Ramadan et al. 2020). It was also shown that cows suffering from abortion and those suffering from downer cow syndrome also exhibit an increase in urea levels (Molefe and Mwanza 2019). The elevated urea levels suggest that the kidneys’ ability to eliminate nitrogenous waste from the bloodstream is compromised (Gayakawad et al. 1999). The measurement of blood urea concentration aids in tracking the body's protein catabolism, which includes the breakdown of urea by bacteria in the rumen; this increase in urea in cows with MFs reflects high ureagenesis (Van Soest 1994). Furthermore, it was revealed that the increase in urea concentration may lead to abortion in cows (Rhoads et al. 2009).

The evaluation of creatinine in the current study reveals no significant differences in the creatinine concentrations between cows with an MF compared with normal pregnant cows. A recent study in cows with metritis showed that there was a smaller drop of creatinine in cows with metritis compared to healthy cows (Cui et al. 2019). The later study suggested that a more severe NEB status may be present in cows with metritis. Similarly, cows with MFs may suffer from severe NEB status.

The most widely used and precise markers of hepatocyte damage are AST and ALT (Thapa and Walia 2007; Puppel and Kuczyńska 2016). TBIL and direct bilirubin (DBIL) are common indicators for liver metabolic capacity (Thapa and Walia 2007; Puppel and Kuczyńska 2016). In our study, although ALT was found to be lower in the mummified group, the other liver enzymes were found to be higher. Increased levels of liver indicators such as AST, ALP and bilirubin were observed in MF group, which was in consistency with the study that mentioned that partum and postpartum AST activities were found at higher levels than in prepartum ones (Cavestany et al. 2005).

It is worth noting that the liver indicators in cows with MFs showed higher values; these findings are consistent with those of studies conducted on cows suffering from metritis, endometritis (Sattler and Fürll 2004) and mastitis (Amany et al. 2008). It is suggested that the mummification of the foetus leads to increased cell membrane permeability and enzyme leakage into the circulation, as the later studies suggested that inflammation can do the same. However, other studies suggested that higher values of liver indicators, when combined with the higher TG levels in the metritis cows (Cui et al. 2019), support the authors’ hypothesis that TG accumulates within the hepatocytes during the postpartum NEB state as a result of the ongoing use of body fat as an energy source, compromising liver function (Goff and Horst 1997). Therefore, in the current study, the same is interpreted to occur in the mummified group, where the liver indicators exhibit higher values combined with higher values of TG level. Moreover, other studies stated the same point of view: Higher AST and ALP levels were strongly linked to liver failure (Voros and Karsai 1987; Lenz 1993) which may also be related to endotoxaemia, hepatic lipidosis and hepatocyte injury (Zadnik 2003; El‐Attar et al. 2008).

An increase in serum GGT activity denotes acute liver injury, and the GGT enzyme is thought to be selective for identifying lesions associated with the hepatic tissue (Bobe et al. 2004). In our study, no difference was discovered in serum GGT concentration between cows with and without an MF. This finding was in the same line with that previously found in cases of endometritis, as GGT concentrations were found to not differ in cows with and without endometritis (Heidarpour et al. 2012; Souza et al. 2010). In contrast, in cows with purulent vaginal discharge (PVD), the enzymatic activity was observed to be marginally greater than in healthy ones (Paiano et al. 2019). The later study indicated that the GGT enzyme activity values in the PVD group would be responsible for some of the hepatocyte damage. Additionally, a recent study revealed that cows with higher GGT after calving had longer postpartum anestrous intervals and lower genetic merit for fertility (Grala et al. 2022). Moreover, fasciolosis, ketosis, angiomatosis and cholestasis are linked to harm to the bile ducts and hepatic cells caused by GGT (Meissonnier and Rousseau 1976).

In the reproductive system, where uterine contractility is crucial for sufficient uterine involution, calcium is a necessary mineral for the induction of this physiological process (Kimura et al. 2002). The current investigation found that the MF‐carrying cows had lower serum Ca and P concentrations than the normal pregnant cows. Previous studies in clinical cases of pyometra and endometritis stated the same results, as the calcium and phosphorus concentrations were found to be lower in the cows with pyometra or endometritis compared to normal healthy control cows (Amin et al. 2021; Heidarpour et al. 2012; Mateus and Lopes da Costa 2002).

In a comparison between cows with normal postpartum plasma Ca concentrations and those with delayed subclinical hypocalcaemia, results revealed that the latter are more prone to illness and yield less milk (McArt and Neves 2020; Serrenho et al. 2021). Moreover, dystocia‐affected cows had lower levels of Ca either in plasma or foetal fluids 24 h after calving than normal‐delivered animals (Benzaquen et al. 2015; Amin et al. 2020).

Decreased levels of Ca can cause uterine atony, which may lead to the failure to expel the MF. This may give an interpretation for the causes of the failure of cows to have an MF by expelling its foetus outside the uterus. Moreover, it was reported that the normal blood Ca level in dairy cows is between 8.5 and 10 mg/dL (Goff 2008). Around calving, there is a marked drop in plasma calcium levels (Goff 2008). During the early stages of lactation, dairy cows usually excrete 30–40 g Ca per day through the production of colostrum (containing 1.7–2.3 g Ca kg^−1^) or milk (containing 1.2 g Ca kg^−1^) (Goff 2004). Lower blood Ca levels cause smooth muscle, particularly the skeletal muscle's sarcoplasmic network, to have less Ca stored in it. Accordingly, in the absence of abdominal muscular contractions and uterine contractions or uterine tiredness, parturition in cattle may go longer than expected, potentially resulting in dystocia and stillbirth (Schuenemann et al. 2011).

Phosphorus is a crucial bone mineral that is involved in many bodily metabolic processes. Prominent inadequacy in reproduction is caused by phosphorus shortages. It primarily modifies and disrupts the oestrous cycle, which results in decreased ovarian activity, irregular ovulation and decreased rates of conception (Amin, Mahmoud et al. 2023). It has previously been shown that cows with retained placentas had decreased serum phosphorus levels (Ray et al. 2004). A low quantity of phosphorus in the pre‐ and post‐calving stages increases the likelihood that the dam will hold onto the foetal membranes (Tillard et al. 2008). Low phosphorus levels may potentially be the cause of the uterine muscles’ diminished ability to contract and result in the failure of an expulsion MF.

Iron is one of the most important minerals that plays a role in different body activities such as haemoglobin formation, thyroid hormone formation, immunological challenges (Cherayil 2010) and ovarian rebound (Kurpińska et al. 2019). Haemoglobin is mostly composed of iron, with four Fe atoms found in each of the four porphyrin rings (Suttle 2010). Blood iron levels are greatly influenced by parturition. Results showed that the level of serum Fe was higher in the mummified group than in the normal pregnancy group. A recent study stated that postpartum blood Fe levels were found to be lower than those during late pregnancy (Mohammed et al. 2021).

Parturition has been shown to dramatically lower serum Fe concentrations due to immune responses and ovarian activity (Kurpińska et al. 2019). As a result, it is important to closely check the blood levels of Fe. However, unless blood loss happens, iron is easily maintained in the body. Although iron deficiency causes a lot of drawbacks for the dam and the foetus as it participates in several other processes, including immunity, energy metabolism and blood production, excessive consumption of Fe should be handled carefully, as it might have antagonistic effects with other trace minerals, including Cu, Mn, Se and Zn.

Magnesium, such as Ca, reduces neuromuscular irritability. Therefore, tetany, or spontaneous muscular spasms, in dairy cows is caused by a decrease in blood magnesium content (Holtenius et al. 2008). It was reported that hypomagnesaemia occurs during the PPP in dairy cows (Neves et al. 2017; Doncel et al. 2019). The findings of the present investigation showed that the Mg level (mg/dL) was within the normal range (2–2.4) (Masoero et al. 2003; Fadlalla et al. 2020) and that the Mg levels were higher in cows with an MF than in normal pregnant cows. The increase in Mg level has been found non‐significant which may be attributed to its tight physiological regulation. Although the increase was non‐significant, these results were in agreement with a recent study conducted in dairy cows that revealed Mg concentration was significantly higher in cows suffering from dystocia and Downer cow syndrome (Molefe and Mwanza 2019). In addition, it was reported that a rise in blood Mg levels is usually observed in cases of decreased Ca levels, as occurs in cases of Downer cow syndrome (Adams et al. 1996) and in the cases of MFs, as seen in the present study.

Maintaining the mother's health during pregnancy and the PPP, as well as the appropriate development of the embryo/foetus and newborn calf, depends on maintaining water‐electrolyte homeostasis. For this reason, it is crucial to keep an eye on variations in the concentration of the major electrolytes found in bodily fluids, such as potassium, sodium and chlorides, as well as the resulting molality of blood plasma (Knight 2001).

One important electrolyte found in extracellular fluid is Na, which is mostly in charge of keeping this fluid's volume and osmotic pressure constant. Cow blood plasma has a Na content that falls between 134 and 156 mmol/L, which is a wide range of reference values (Winnicka 2008). Numerous publications claim that in healthy adult cows, Na balance is effectively regulated and blood salt concentration is maintained within relatively small ranges. However, significant variances may be seen based on the physiological state or stage of life. Our study on the mummified group showed that their blood had a higher Na concentration than the group that was not mummified during a normal pregnancy. However, these changes were statistically insignificant. These findings almost coincide with the results of a recent study that observed that an increase in Na concentration was found in the last week of pregnancy, and the highest concentration was noted on the day of delivery.

Approximately 98% of the cations in intracellular fluid are potassium. The maintenance of intracellular osmotic pressure is attributed to potassium. Additionally, it participates in various systemic functions such as preserving the acid–base balance, transmitting nerve signals, muscle contraction and relaxation, activating numerous enzymes, glycogen production and protein synthesis (Jain et al. 2007; Hassabo 2008). According to reports, the concentration of potassium in cows’ blood is steady between 3.8 and 5.1 mmol/L during pregnancy and the start of lactation (Winnicka 2008). Our results on mummified group showed a significant difference in potassium concentration, which was higher than that of normal pregnant group and was higher than the normal range. However, a previous study illustrated that in the second trimester of pregnancy, potassium concentration was recorded to be at its lowest levels compared to the third trimester, in which it was at the highest levels (Yokus and Cakir 2006). This may give an explanation for the rise in potassium concentration in mummified group that is supposed to end the pregnancy and expel the foetus.

Extracellular fluid is primarily rich in chlorides. The range of chloride concentrations in blood serum in healthy cows is 93–107 mmol/L (Winnicka 2008). Our results on mummified group showed an increase in chloride concentration, which was non‐significant compared to normal pregnant group. Blood chloride concentration and Na concentration are regulated together, which implies that variations in Na concentration can impact variations in chloride concentration (Batchelder et al. 2007; Ożgo et al. 2008).

Despite changes in electrolyte concentration, the relative stability of blood osmotic pressure during the final month of pregnancy and the first month of lactation shows how effective the mechanisms regulating osmotic stability and electroneutrality of extracellular fluid are (Skrzypczak et al. 2009). Vasopressin concentration rises in response to any increase in blood plasma osmotic pressure, peaking during delivery (Landgraf et al. 1983). Additionally, starting 30 days prior to birth, Ożgo et al. (2008) showed an increase in plasma renin activity and an increase in vasopressin concentration in the blood. Vasopressin and V2R agonists not only increase the water permeability of the renal collecting duct (CD) but also stimulate Na reabsorption (Schafer and Hawk 1992; Schafer 2002). Thus, hyper‐vasopressin‐dependent activation of the epithelial Na channel (ENaC) may be associated with an increased risk of blood pressure and Na retention. This may give an explanation for the rise in water‐electrolyte (Na, K and chloride) concentrations in mummified group that are supposed to end the pregnancy, get ready for delivery and expel the foetus.

Oxytocin also has a role in controlling osmotic pressure. The frequency and amplitude of oxytocin secretion increase prior to birth (Williams et al. 2001). When atrial natriuretic peptide is released in response to oxytocin, it facilitates the excretion of water and electrolytes, which raises the levels of water and electrolytes (Na, K and chloride) (Somponpun and Sladek 2003).

## 5 Conclusion

The current study showed that the group of pregnant cows suffering from MFs exhibited a significant reduction in some hormones, such as T3 and T4. In addition, the biochemical profile indicates the presence of a significant decrease in GLU and albumin compared with normal pregnant animals. In contrast, TG, cholesterol, globulin and TP were significantly higher in MFs group. Kidney and liver profiles showed a significant increase in urea, AST, ALP and bilirubin and a significant decrease in ALT in cows with an MF compared with normal pregnant cows. Mineral profiles showed a significant decrease in Ca and P in mummified group compared to normal pregnancy group. All of the above reveals that cows with MFs maintained variant biological changes in the body. These findings can be used as an indicator for cow health and as a means of diagnosis to prevent pregnancy abnormalities that arise in the later stages of pregnancy and the PPP.

## Author Contributions

**Yahia A. Amin** and **Ragab H. Mohamed**: conceptualization. **Yahia A. Amin**: data curation. **Yahia A. Amin**, **Obeid Shanab**, **Foad Farrag** and **Eman M. Abu El‐Naga**: formal analysis. **Yahia A. Amin**, **Obeid Shanab**, **Foad Farrag**, **Ahmed A. Elolimy**, **Mustafa Shukry**, **Mohamed Abdelmegeid**, **Eman M. Abu El‐Naga** and **Ragab H. Mohamed**: funding acquisition. **Yahia A. Amin**, **Ibrahim S. Zahran**, **Mariam A. Fawy**, **Eman M. Abu El**‐**Naga** and **Ragab H. Mohamed**: investigation. **Yahia A. Amin** and **Ragab H. Mohamed**: methodology. **Yahia A. Amin**: project administration**. Yahia A. Amin** and **Ragab H. Mohamed**: resources. **Yahia A. Amin**, **Ibrahim S. Zahran**, **Mariam A. Fawy**, **Ahmed A. Elolimy**, **Mustafa Shukry** and **Eman M. Abu El‐Naga**: software. **Yahia A. Amin**: supervision. **Yahia A. Amin** and **Obeid Shanab**: validation. **Yahia A. Amin** and **Ragab H. Mohamed**: visualization. **Yahia A. Amin**: writing – original draft. **Yahia A. Amin**: writing – review and editing.

## Ethics Statement

Experimental procedures were approved by the Ethical Research Committee members of the Faculty of Veterinary Medicine of South Valley University, Qena, Egypt (No. 96/03.11.2022).

## Conflicts of Interest

The authors declare no conflicts of interest.

### Peer Review

The peer review history for this article is available at https://www.webofscience.com/api/gateway/wos/peer‐review/10.1002/vms3.70304.

## Data Availability

All data related to this research were illustrated in the manuscript.
